# Dissection of the Role of PfEMP1 and ICAM-1 in the Sensing of *Plasmodium falciparum*-Infected Erythrocytes by Natural Killer Cells

**DOI:** 10.1371/journal.pone.0000228

**Published:** 2007-02-21

**Authors:** Myriam Baratin, Sophie Roetynck, Bruno Pouvelle, Céline Lemmers, Nicola K. Viebig, Sofia Johansson, Philippe Bierling, Artur Scherf, Jürg Gysin, Eric Vivier, Sophie Ugolini

**Affiliations:** 1 Aix-Marseille Université, Faculté des Sciences de Luminy, Centre d'Immunologie de Marseille-Luminy (CIML), Marseille, France; 2 Institut National de la Santé et de la Recherche Médicale (INSERM), U631, Marseille, France; 3 Centre National de la Recherche Scientifique (CNRS), UMR6102, Marseille, France; 4 Unité de Parasitologie Expérimentale, Université de la Méditerranée, Marseille, France; 5 Institut Pasteur and Centre National de la Recherche Scientifique (CNRS), Paris, France; 6 Etablissement français du sang, Créteil, France; 7 Hôpital de la Conception, Assistance Publique – Hôpitaux de Marseille, France; Federal University of São Paulo, Brazil

## Abstract

**Background:**

Host innate immunity contributes to malaria clinical outcome by providing protective inflammatory cytokines such as interferon-γ, and by shaping the adaptive immune response. *Plasmodium falciparum (Pf)* is the etiologic agent of the most severe forms of human malaria. Natural Killer (NK) cells are lymphocytes of the innate immune system that are the first effectors to produce interferon-γ in response to *Pf*. However, the molecular bases of *Pf*-NK cell recognition events are unknown. Our study focuses on the role of *Pf* erythrocyte membrane protein 1 (PfEMP1), a major *Pf* virulence factor. PfEMP1 is expressed on parasitized-erythrocytes and participates to vascular obstruction through the binding to several host receptors. PfEMP1 is also a pivotal target for host antibody response to *Pf* infection.

**Methodology/Principal Findings:**

Using genetically-engineered parasite mutant strains, a human genetic deficiency, and blocking antibodies, we identified two receptor-ligand pairs involved in two uncoupled events occurring during the sensing of *Pf* infection by NK cells. First, PfEMP1 interaction with one of its host receptor, chondroitin sulfate A, mediates the cytoadhesion of *Pf*-infected erythrocytes to human NK cell lines, but is not required for primary NK cell activation. Second, intercellular adhesion molecule-1 (ICAM-1), another host receptor for PfEMP1, is mandatory for NK cell interferon-γ response. In this case, ICAM-1 acts via its engagement with its host ligand, LFA-1, and not with PfEMP1, consistent with the obligatory cross-talk of NK cells with macrophages for their production of interferon-γ.

**Conclusion/Significance:**

PfEMP1-independent but ICAM-1/LFA-1-dependent events occurring during NK cell activation by *Pf* highlight the fundamental role of cellular cooperation during innate immune response to malaria.

## Introduction

*Plasmodium falciparum* (*Pf*) causes the most severe forms of malaria. Experimental and natural *Pf*-infection studies suggest that an early IFN-γ production is pivotal to the clinical outcome of the disease [1]. NK cells have been characterized by their role in the defense against tumor, allogeneic and microbe-infected cells [2], and have also been recently identified as a major source of IFN-γ within human PBMC exposed to *Pf*-infected erythrocytes (IE) *in vitro* [3], [4], [5]. Although direct recognition of IE by NK cells induce functional responses such as CXCL8 production [4], we have shown that NK cell IFN-γ production in response to IE was highly dependent on the cooperation with monocytes/macrophages [4]. Similar results, involving myeloid accessory cells, have been recently reported [6]. Myeloid cell-derived interleukin (IL)-18 and IL-12 were shown to be mandatory for NK cell IFN-γ response [4], [6], [7]. However, the innate receptors engaged in the interactions between IE, NK cells and macrophages remain to be identified. Toll like receptor (TLR) 2 was reported to recognize glycosylphosphatidyl inositol (GPI) anchors of *Pf* [8], but our previous studies ruled out a requirement for this molecule in NK cell activation [4].

*Pf* infection results in the exposure at the red blood cell (RBC) surface of multiple molecules [9] that could be candidate ligands involved in NK cell activation. Among them, PfEMP1 molecules, belong to a large family of parasite proteins that have been extensively characterized for their capacity to mediate IE cytoadhesion to the vascular endothelium [10]. This adhesion process, often described as a way for the parasite to evade splenic clearance, has also been reported as associated to vascular obstruction of vital organs leading to major pathological events. PfEMP1 proteins are encoded by ∼60 different *var* genes, but deletions, recombinations and gene conversions create a virtually unlimited repertoire [11]. *var* gene expression is mutually exclusive such that only a single copy is transcribed in one parasite at any one time [12]. Binding experiments using recombinant PfEMP1 domains have shown an interaction with several host receptors [13]. Three of them, CD36, ICAM-1 and chondroitin sulfate A (CSA) have been extensively studied. ICAM-1 is expressed on hematopoietic cells and interacts with the β2 integrins CD11a/CD18 (LFA-1), CD11b/CD18 (Mac-1) or CD11c/CD18, which are involved in cell adhesion and activation [14]. ICAM-1 is also a co-stimulatory receptor that promotes T cell activation and initiates intracellular signaling [15]. CSA is a sulfated glycosaminoglycan chain with a vast structural diversity that decorates several cell surface molecules [16]. CSA has been involved in pregnancy-associated malaria, a severe form of the disease due to massive sequestration of the parasite in the placenta. Along this line, most *Pf* strains isolated from women suffering of pregnancy-associated malaria cytoadhere to CSA [17]. Finally, CD36 is a broadly expressed class B scavenger receptor which supports adherence of most *Pf* natural isolates [18]. CD36 has been involved in the non opsonic phagocytosis of IE by macrophages [19], and in the modulation of monocyte-derived dendritic cells function by IE [20]. Mouse CD36 is also a co-receptor for TLR2 and may thus act as an activating molecule [21]. Altogether these data prompted us to investigate the role of PfEMP1, CSA, CD36 and ICAM-1 in the direct and indirect detection of IE by NK cells.

## Results and Discussion

### CSA is involved in the cytoadhesion of IE to NK cell lines but is dispensable for primary NK cell activation

**Figure 1:**
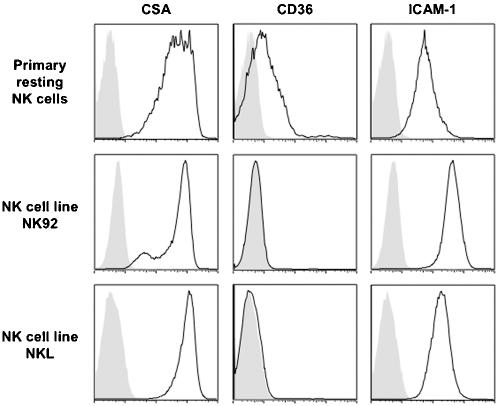
Expression of CSA, CD36 and ICAM-1 on human NK cells. Primary resting human NK cells (CD56^+^CD3^−^) in total PBMC as well as human NK cell lines, NK92 and NKL were analyzed by flow cytometry to determine the surface expression of three host ligands for PfEMP1: CSA (left panels, dark line), CD36 (middle panels, dark line) and ICAM-1 (right panels, dark line). Stainings with isotype control for each antibody are represented by filled grey histograms. Stainings of primary resting human NK cells are representative of at least 3 donors.

Physical interactions between NK cells and IE have been described upon co-culture [4], [22]. Since PfEMP1 is responsible for cytoadhesion of IE to endothelial cells, we hypothesized that this cell surface parasite protein might also play a role in IE detection by NK cells. Flow cytometry analysis revealed that circulating NK cells expressed three major receptors for PfEMP1: CSA, CD36 and ICAM-1 (Fig. 1). The human NK cell lines, NK92 and NKL, also expressed CSA and ICAM-1 but not CD36 (Fig. 1).

**Figure 2:**
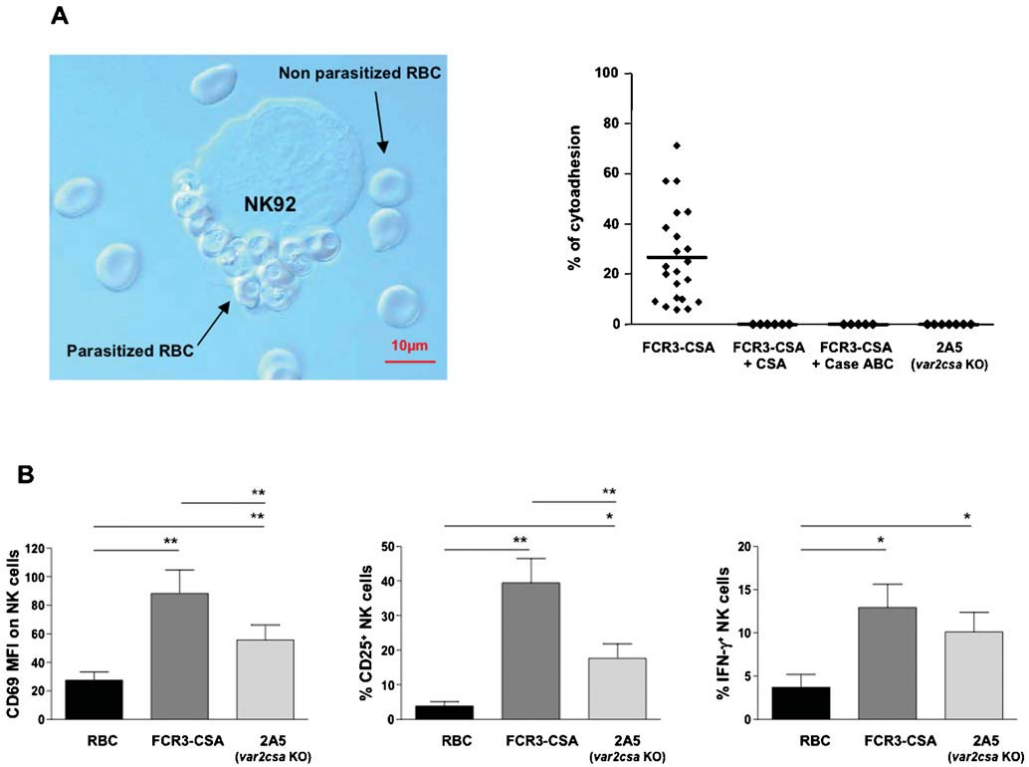
CSA is involved in IE interaction with human NK cell line but not in primary NK cell activation. (A) The human NK cell line NK92 was incubated with whole culture of IE in different conditions. After 1 h at 37°C, a sample of the co-culture was placed between slide and cover and analyzed under a microscope. The NK92 cells directly interacts with IE (FCR3-CSA strain) as rosettes, but not with uninfected erythrocytes (x100 original magnification, left panel). RBC infected with FCR3-CSA or 2A5 were co-cultured with NK92 cells alone or in the presence of soluble CSA (+CSA), or with NK92 cells pre-treated with chondroitinase ABC (+Case ABC). At the end of the co-culture, the percentage of NK cells interacting with at least two IE was determined and expressed as % of cytoadhesion. Each dot represents one independent experiment (right panel). (B) Freshly isolated human PBMC were cultured with uninfected RBC (RBC), RBC infected with FCR3-CSA or RBC infected with the *var2csa* KO parasite 2A5. After 24 h, NK cell activation was analyzed by flow cytometry by gating on CD3^−^CD56^+^ lymphocytes. The CD69 MFI (mean fluorescence intensity) staining on NK cells (left panel), the percentage of CD25^+^ NK cells (middle panel) and the percentage of IFN-γ^+^ NK cells (right panel) were determined for 7 different healthy donors. Means±SEM are represented. Statistical analyses were performed using the Wilcoxon test.

We first evaluated the involvement of CSA in the direct detection of IE by NK cells, using the FCR3-CSA parasite strain that has been selected by panning to adhere to CSA [23]. Upon co-culture, FCR3-CSA-infected RBC rapidly adhered to NK92 cells forming structures reminiscent of rosettes (Fig. 2A, left panel). Rosettes were defined as conjugates engaging one NK cell bound to at least two IE. This striking phenotype was specific of parasitized RBC because uninfected RBC did not form rosettes with NK cells. Quantification revealed that after 1 h of co-culture 26.7%±4 NK92 cells formed rosettes with FCR3-CSA-infected RBC (Fig. 2A, right panel). This interaction was totally abrogated when NK92 cells were pre-treated with chondroitinase ABC that degrades CSA or when an excess of soluble CSA was added in the co-culture (Fig. 2A, right panel). Adding soluble CSA on preformed rosettes disrupted these structures and similar results were obtained with NKL cells (data not shown). To definitively demonstrate the role of CSA in the formation of rosettes between NK cells and IE, we used the 2A5 strain, a FCR3 mutant in which the *var2csa* gene is disrupted [24]. This PfEMP1- encoding gene was shown to be essential for the binding to CSA [24]. RBC infected by 2A5 were unable to form rosettes with NK92 cells (Fig. 2A, right panel). Thus PfEMP1 that binds CSA acts as a dominant molecule in the cytoadhesion of IE to human NK cell lines.

No stable rosettes between primary resting NK cells and CSA-binding IE were detected. Since the CSA sugar chain length and sulfate content is critical for the binding of PfEMP1 [25], it is possible that peripheral NK cells do not express the appropriate CSA structure. However subsets of NK cells, such as activated NK cells or uterine NK cells which accumulate in the placenta during pregnancy [26], [27], might express the CSA features required for rosette formation.

Although IE did not form rosette with primary resting NK cells, we observed conjugates that involved one NK cell bound to one IE. Consistent with previous data [22], these cellular contacts were visualized with several different *Pf* strains including CSA-adhesive strains (data not shown). We thus investigated whether CSA was involved in the activation of primary NK cells. After 24 hours co-incubation of PBMC with FCR3-CSA-infected RBC, NK cells up-regulated CD69, CD25 at their surface and produced IFN-γ (Fig. 2B). Importantly, NK cells were also responsive to RBC infected with the 2A5 strain that is deficient for the binding to CSA. As shown in Figure 2B, IFN-γ production by NK cells following PBMC stimulation with 2A5-infected RBC was very similar to the one induce by the parental strain even though CD69 and CD25 up-regulation was significantly lower (Fig. 2B). These data suggest that although the interaction between CSA and PfEMP1 might contribute to NK cell activation, it was not mandatory.

### NK cells deficient in CD36 expression are reactive to IE

**Figure 3:**
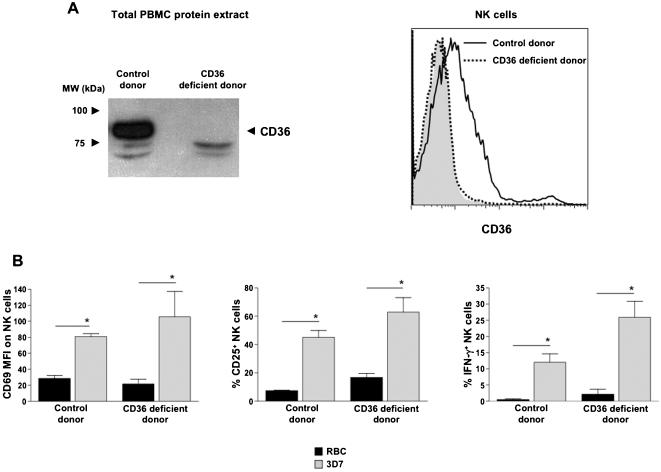
Deficiency in CD36 does not alter NK cell response to IE. (A) The level of CD36 expression was determined on PBMC collected from a healthy donor (Control donor) or from a patient deficient for CD36. Total PBMC protein extracts were prepared and analyzed by Western Blot (left panel). Total PBMC were stained with CD36 antibody or with an isotype control (filled grey histogram, right panel). Histograms for CD3^−^CD56^+^ NK cells from a CD36 deficient donor (dotted line) and a control donor (bold line) are represented. (B) Control or CD36-deficient PBMC were cultured with uninfected RBC (RBC, black bars), or with RBC infected with the 3D7 *Pf* strain (3D7, grey bars). After 24 h, NK cell activation was analyzed by flow cytometry by gating on CD3^−^CD56^+^ NK cells. The CD69 MFI staining on NK cells (left panel), the percentage of CD25^+^ NK cells (middle panel) and the percentage of IFN-γ^+^ NK cells (right panel) were determined in three independent experiments. Means ± SEM are represented. Statistical analyses were performed using the Mann Whitney test.

We next explored the role of CD36 in NK cell activation by IE. We took advantage of the existence of CD36 genetic deficiencies in the human population [28]. PBMC were isolated from a CD36-deficient patient and the absence of CD36 was confirmed by immunoblot analysis as well as by flow cytometry (Fig. 3A). The 3D7 parasite was chosen in the following settings, because it is an oligoclonal laboratory strain that is not selected for any adhesive phenotype, and it is one of the most potent *Pf* strain for NK cell activation [4]. Remarkably, CD36-deficient NK cells within PBMC were as reactive as normal NK cells to 3D7-infected RBC (Fig. 3B). Therefore PfEMP1:CD36 interaction was dispensable for NK cell activation by IE. Since the patient included in our study did not express CD36 on any PBMC, our data also showed that CD36 was neither required for the direct sensing of IE by NK cells nor for the suitable activation of macrophages, their obligatory partners.

### NK cell IFN-γ production requires engagement of ICAM-1 with LFA-1, but not with PfEMP1

**Figure 4:**
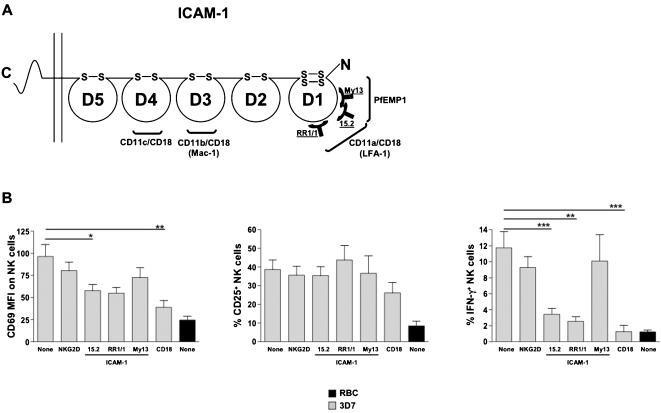
Engagement of ICAM-1 with its cellular ligand but not with PfEMP1 is required for NK cell IFN-γ production. (A) Diagram of an ICAM-1 molecule showing schematic binding sites for LFA-1, Mac-1, CD11c/CD18 and PfEMP1. The epitope map of the anti-ICAM-1 mAb 15.2, My13 and RR1/1 is indicated. (B) Human PBMC were cultured with uninfected RBC (RBC, black bars) or with RBC infected with the 3D7 *Pf* strain (3D7, grey bars) in presence or absence of antibodies directed against NKG2D (isotype control), ICAM-1 or CD18. Three different clones of anti-ICAM-1 were used: 15.2 blocks the interaction of ICAM-1 with LFA-1 and with PfEMP1, RR1/1 blocks only the interaction with LFA-1 and My13 blocks only the interaction with PfEMP1. After 24 h of co-culture, NK cell activation was analyzed by flow cytometry by gating on CD3^−^CD56^+^ NK cells. The CD69 MFI staining on NK cells (left panel), the percentage of CD25^+^ NK cells (middle panel) and the percentage of IFN-γ^+^ NK cells (right panel) were determined for 24 donors (None, NKG2D and 15.2), 13 donors (CD18), 9 donors (My13) or 5 donors (RR1/1). Means ± SEM are represented. Statistical analyses were performed using the Mann Whitney test.

We next addressed the role of ICAM-1, a third major PfEMP1 receptor, in NK cell activation by IE. We first assessed the NK cell response to IE, in the presence or absence of the blocking monoclonal antibody (mAb) 15.2 directed against ICAM-1 [29] (Fig. 4A). Addition of the mAb 15.2 to the co-culture drastically impaired IFN-γ production by NK cells and significantly reduced the CD69 up-regulation (Fig. 4B, right and left panels). In contrast CD25 up-regulation remained unchanged in the presence of the mAb (Fig. 4B, middle panel). An isotype control mAb directed against the NK cell surface receptor, NKG2D, did not alter the NK cell response (Fig. 4B). Thus ICAM-1 engagement is critical for NK cell IFN-γ production and participates to CD69 up-regulation. However CD25 up-regulation is independent of ICAM-1, indicating that NK cell activation by IE involve at least two different pathways.

ICAM-1 consists of five Ig-like domains (D1 to D5) a short transmembrane region and a small carboxyl terminal cytoplasmic domain [30] (Fig. 4A). The PfEMP1 and LFA-1 binding sites overlap and are located into the D1 domain of ICAM-1 whereas its two other host ligands, CD11c/CD18 and Mac-1 bind respectively to domains D4 and D3 [31], [32]. The mAb 15.2 inhibits both the PfEMP1:ICAM-1 and the LFA-1:ICAM-1 interactions [29], [33]. Thus, to independently address the role of LFA-1 and PfEMP1 in NK cell response to IE, we used two other anti-ICAM-1 blocking mAb directed against distinct epitopes. The mAb RR1/1 only blocks the LFA-1:ICAM-1 interaction and the mAb My13 only inhibits the binding of ICAM-1 to PfEMP1 [29] (Fig. 4A). The addition of RR1/1 to the co-culture mimicked the effect of 15.2, showing a mandatory role of the interaction between ICAM-1 and LFA-1 in NK cell IFN-γ response to IE (Fig. 4B, right panel). Of note, IE and non infected erythrocytes do not express ICAM-1 or LFA-1 (data not shown), excluding a role of these two molecules at the surface of RBC. We next confirmed the importance of the interaction between ICAM-1 and its host ligand LFA-1 by showing that TS1/18, a blocking mAb directed against the β2 subunit (CD18) of LFA-1, inhibited NK cell IFN-γ production and reduced CD69 up-regulation in the same way than 15.2 (Fig. 4B, right and left panels). In contrast, the anti-ICAM-1 blocking mAb, My13, had no impact on NK cell response (Fig. 4B), ruling out a major role of an interaction between PfEMP1 and ICAM-1. Although the cell surface expression of CD69 is increased on purified NK cells in the presence of IE, the extent of this up-regulation is lower than when NK cells are included in PBMC, i.e., when they cooperate with macrophages [4]. The inhibition of CD69 up-regulation by anti-ICAM-1 mAb is thus consistent with the role of ICAM-1:LFA-1 interaction in the helper function of macrophages to NK cell activation induced by IE. Therefore, an ICAM-1:LFA-1-dependent pathway promotes both CD69 up-regulation and IFN-γ secretion by NK cells in the presence of IE. As macrophages and NK cells express both ICAM-1 and LFA-1, it is possible that bidirectional interactions involving these molecules are required for optimal NK cell response. In contrast, the NK cell CD25 up-regulation induced by IE is independent of ICAM-1:LFA-1 interactions.

### PfEMP1 deficient parasites are efficient activators of NK cells

**Figure 5:**
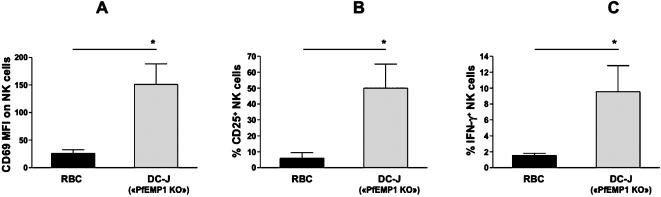
PfEMP1 deficient parasites are potent activators of NK cells. Human PBMC were cultured with uninfected RBC (RBC, black bars) or with RBC infected with the “PfEMP1 KO” strain DC-J. After 24 h, NK cell activation was analyzed by flow cytometry by gating on CD3^−^CD56^+^ NK cells. The CD69 MFI staining on NK cells (left panel), the percentage of CD25^+^ NK cells (middle panel) and the percentage of IFN-γ^+^ NK cells (right panel) were determined for 5 different healthy donors. Means±SEM are represented. Statistical analyses were performed using the Wilcoxon test.

Our results showed that neither CSA, nor CD36, nor ICAM-1 was required as PfEMP1 receptor for NK cell activation. However, due to the high diversity of the *var* genes, we could not exclude a role of any other PfEMP1 molecule in NK cell activation by IE. To address this issue, we used a mutant parasite strain deficient for PfEMP1 expression; DC-J is a transgenic parasite selected to express a *var* gene in which the PfEMP1 coding region has been replaced by a drug selectable marker leading to the silencing of all other *var* genes in the *Pf* genome [34]. In addition, DC-J parasites lack *kahrp* (knob-associated histidine-rich protein) (data not shown), which is essential for the formation of electron dense protrusions at the surface of IE called knobs [35]. Remarkably, NK cells were still responsive to DC-J-infected RBC as they up-regulated CD69 and CD25, and produced IFN-γ upon co-culture (Fig. 5). Altogether these results ruled out a mandatory role for any PfEMP1 molecules in NK cell activation and indicated also that knobs were not essential structures for NK cell response to IE.

### Concluding remarks

The prediction of the host response to microbes necessitates the precise dissection of innate recognition mechanisms [36], [37]. We identified here two receptor:ligand pairs involved in two uncoupled events occurring during the sensing of *Plasmodium* infection by NK cells; PfEMP1:CSA interaction mediates cytoadhesion of IE with NK cells and ICAM-1:LFA-1 interactions play a major role in the cellular cooperation required for NK cell IFN-γ response. In addition to these two modes of IE sensing, our data revealed a third pathway that leads to CD25 up-regulation in a PfEMP1 and ICAM-1 independent pathway.

These different components of the NK cell response may lead to differential outcomes in the physiopathology of malaria. Indeed, massive sequestration of CSA-binding parasites in the placenta leads to severe clinical malaria in primigravid women [17]. CSA-binding IE are rarely observed outside of pregnancy and primigravid women generally lack acquired immunity to these parasites, suggesting that CSA-binding IE are rapidly eliminated from the host [38]. As NK cells traffic through the spleen, conjugate formation with CSA-binding IE, might contribute to parasite splenic destruction. On the other hand, during the first part of normal pregnancy, NK cells invade the decidua [39], so that they might be used by the parasite as selective uterine carriers. It is thus tempting to speculate that the potential rosetting of IE with activated NK cells through PfEMP1:CSA interaction may serve as a *Pf* dissemination strategy. This hypothesis that would reveal a selective advantage of *Pf* *var2csa* gene upon pregnancy remains to be tested by the detection of NK-IE rosettes in spleen and/or in placenta sections obtained from *Pf*-infected individuals.

The second pathway described here relies on the engagement of ICAM-1 with LFA-1 but not with the parasite protein PfEMP1. This essential interaction that is reminiscent of the obligate cross-talk between NK cells and myeloid accessory cells revealed that in addition to soluble factors these two partners cooperate through cellular contacts. Interestingly, an ICAM-1 allelic variant termed ICAM-1^kilifi^ has been identified in malaria endemic area. This polymorphism is highly frequent in sub-saharian Africa, but is not found in Caucasian populations [40]. While some genetic analyses have associated ICAM-1^kilifi^ to cerebral malaria susceptibility, other studies suggested a protective role of this mutation or did not support any correlation [40]. ICAM-1^Kilifi^ was not only shown to alter the interaction with some Pf strains in adhesion assays but also to exhibit a reduced avidity for LFA-1 as compared to the commonly expressed ICAM-1 allele [41]. Our data suggest that the role of ICAM-1 polymorphism should thus be revisited in light of its potential implication during the early NK cell response to Pf infection. Along this line, the interplay between the effect of ICAM-1^Kilifi^ on innate immune response and, on parasite sequestration may help to explain the apparently conflicting reports on the association of ICAM-1^kilifi^ with severe malaria.

## Materials and Methods

### Parasites

*Pf* were cultured as previously described [4] and were routinely proven to be mycoplasma-free by PCR (kit from Minerva Biolabs). All the strains used in this study are oligoclonal laboratory strains selected for the required characteristic; FCR3-CSA parasites were selected by panning to ensure that all of them adhere to CSA [23]. 2A5 is a var2csa disruption mutant of FCR3 that is unable to recover the CSA-binding phenotype [24]. It was cultured with the selective drug WR99210 at 2.5 nM. DC-J is a transgenic parasite selected to express a var gene in which the PfEMP1 coding region has been replaced by a drug selectable marker leading to the silencing of all other var genes in the genome [34]. DC-J mutant parasites were maintained in culture with blasticidin S HCL at 20 µg/ml. Cultures of trophozoites and mature schizont-infected erythrocytes (IE) were enriched to 60–90% using Plasmion or Percoll gradient.

### Antibodies and reagents

PerCP-Cy5.5-CD3 (clone SK7), FITC-CD69 (clone FN50), PE-IFN-γ (clone 4S.B3) mAb were from BD Biosciences. APC-CD56 (clone NHK-1), PE-CD25 (clone B1.49.9) and FITC-CD3 (clone UCHT1) mAb were from Immunotech, Beckman Coulter. Mouse anti-human CD36 mAb, clone FA6-152 (for cytometry) and goat anti-mouse IgG H+L coupled to PE were from Immunotech. Biotin-conjugated rabbit anti-human CD36 (for Western Blot) was from Novus Biologicals. Mouse anti-CSA (BE-123, IgG1κ) was from Chemicon. Streptavin-HRP was from Sigma. Azide free anti-human CD18 mAb (clone TS1/18, Endogen), anti-human ICAM-1 (clone 15.2, Serotec; clone RR1/1, Alexis Biochemicals; clone My13, Zymed Laboratories) and anti-human NKG2D (Clone ON 72) were used at 10 µg/ml. Purified mouse IgG from Zymed were used as isotype control.

### Flow cytometry analysis

Cells were stained for 30 min at 4°C with appropriate combinations of labeled mAb and fixed in 2% paraformaldehyde in PBS. For intracytoplasmic staining, fixed cells were permeabilized and stained with anti-IFN-γ mAb, by using the cytofix/cytoperm kit according to the manufacturer's instructions (BD Biosciences). CSA staining requires chondroitinase digestion by incubating PBMC with 1 U/ml chondroitinase ABC at 37°C for 1 h.

### Cytoadhesion experiments

1×10^6^ NK92 cells were incubated with whole cultures of IE (10×10^6^) in RPMI 1640 at 37°C for 1 h under continuous shaking. When mentioned, soluble CSA was added to the culture at 100 µg/ml. For chondroitinase ABC cell digestion, NK cells were incubated with 1 U/ml of enzyme at 37°C for 1 h. The adhesion of IE on NK cells was observed under an inverted microscope. The percentage of NK cells interacting with IE was determined as the percentage of cytoadhesion.

### PBMC stimulation

PBMC from healthy volunteers (Etablissement Français du Sang) and CD36-deficient individual [28] were isolated by Ficoll gradient according to the local ethics committee on human experimentations. PBMC (5×10^5^) were cultured with IE or RBC (2×106) and after 20 h of culture, CD3^−^CD56^+^ NK cell activation was evaluated by cell-surface expression of CD25 and CD69 as well as by intracellular IFN-γ production [4]. For intracellular IFN-γ assay, Golgi stop (BD Biosciences) was added during the last 6 h of culture.

### Immunoblotting

Whole cell extracts were prepared from total PBMC using hypertonic buffer. After boiling each sample (corresponding to 2.5×106 PBMC) was loaded onto an 8% acrylamide gel. After transfer, the membrane was incubated with biotinylated anti-human CD36 and with Streptavidin-HRP. Probed proteins were detected with the ECL system (Amersham).

### Statistical analysis

All the data were analyzed using the Mann Whitney or the Wilcoxon tests (see figure legends) with the GraphPad Prism software. A value of p≤0.05 was considered significant. Data are represented as mean±SEM. * p≤0.05, ** p≤0.005, *** p≤0.0001
